# “Recurrent Papillary Necrosis and Nephrocalcinosis Induced by Nonsteroidal Anti-Inflammatory Drugs for Gouty Arthritis Associated with Congenital Chloride-Losing Diarrhea: A Major Risk for Kidney Loss”

**DOI:** 10.1155/2021/3558278

**Published:** 2021-10-16

**Authors:** Kamel El-Reshaid, Shaikha Al-Bader, Hossameldin Sallam

**Affiliations:** ^1^Department of Medicine, Faculty of Medicine, Kuwait University, Kuwait City, Kuwait; ^2^Department of Medicine, Nephrology Unit, Al-Amiri Hospital, Ministry of Health, Kuwait City, Kuwait

## Abstract

Congenital chloride-losing diarrhea (CCLD) is a rare genetic disorder due to autosomal recessive mutation in the SLC26A3 gene on chromosome 7. It is characterized with chronic watery diarrhea with high fecal chloride (Cl: >90 mmol/L), low potassium (K), and metabolic alkalosis with low urinary Cl and K. The overall long-term prognosis is favorable with optimal life-long salt and K supplementation. In this case report, we describe a man with progressive renal failure and small kidneys that showed nephrocalcinosis and papillary necrosis. His disease was diagnosed since birth and was confirmed by our tests. He was incompliant with therapy and had developed gout. The latter complication of his disease has led to excessive NSAID use over the past years. Reinstitution of diet, drug therapy, and allopurinol had stabilized his renal disease for 1 year of follow-up. In conclusion, excessive analgesic use is a risk factor for renal failure in CCLD.

## 1. Introduction

Congenital chloride-losing diarrhea (CCLD) is a rare genetic disorder due to autosomal recessive mutation in the SLC26A3 gene on chromosome 7. The latter leads to defective intestinal chloride-bicarbonate exchanges, in epithelial sodium (Na)/hydrogen (H) transport through the Na/H exchangers (NHE2 and/or NHE3), with the resultant intestinal loss of chloride and retention of bicarbonate [1]. Consequently, the renin-angiotensin-aldosterone system is activated with Na reabsorption, potassium (K) excretion, and hypokalemia [2]. The first description of the disease was in 1945 by Gamble and Darrow, and nearly 250 cases have been reported to date [3]. Most of the cases have been reported from Finland, and their calculated prevalence was 1 in 43,000 newborns [4]. In Arab, only few cases have been reported in Saudi Arabia and Kuwait [5, 6]. The overall long-term prognosis is favorable with optimal life-long salt and K substitution though complications such as renal disease, hyperuricemia, inguinal hernias, spermatoceles, and decreased fertility reported in incompliant patients [7]. In this case report; we describe an adult patient, with this rare disease, who lost his kidneys due to chronic analgesic abuse for maltreated gouty arthritis.

## 2. The Case

**Figure 1:**
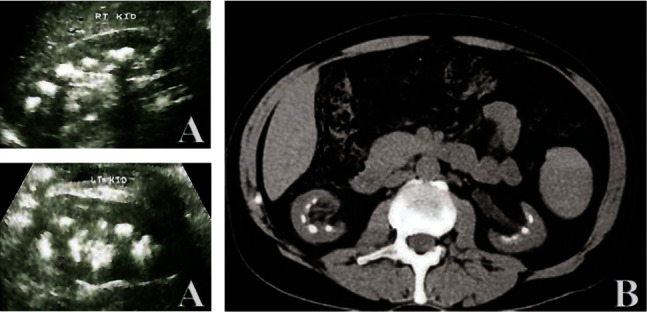
Ultrasound showing a small right kidney with nephrocalcinosis and calcified papilla (a) that was confirmed by CT scan (b).

A 30-year-old man presented with progressive nausea and vomiting over a few weeks. His past medical history revealed watery diarrhea since the age of 6 months. His investigations at that time have shown high stool chloride (Cl) >90 mmol/L with low serum and urinary Cl, potassium (K) and high uric acid (UA). Moreover, he had metabolic alkalosis. He had received high-salt diet in addition to slow K and calcium tablet. Twelve years ago, he had developed recurrent attacks of joint pains with high uric acid which was treated with multiple NSAIDs. On his initial presentation, the patient was conscious, oriented X3, and without distress of shortness of breath or pain. Blood pressure was 180/110 mmHg. He was afebrile with a body weight of 104 kg. He did not have jugular venous distension and oedema. Systemic examination did not show abnormality including lung auscultation. Laboratory investigations showed normal peripheral leucocytic and platelets counts. Hemoglobin was 104 g/L with normal MCV, transferrin saturation%, and vitamin B12. Serum urea and creatinine were elevated at 40 umol/L and 650 umol/L, respectively. Serum electrolytes revealed low sodium, Cl, calcium, and K at 129, 93, 1.8, and 2.9 mmol/L, respectively. Serum UA was elevated at 700 umol/L. Liver tests were normal with serum albumin at 41 g/L. Serum cholesterol and TSH were normal. Serum pH was 7.5, and bicarbonate was at 32 mmol/L. Urine routine and microscopy showed 1(+) proteinuria without hematuria and pyuria. Serum complements (C3 and C4), IgA level, and protein electrophoresis were normal. Anti-CCP, ANA, anti-ds DNA, ANCA, anti-GBM-antibodies, hepatitis B surface antigen, and anti-HCV antibodies were negative. Stool tests were negative for ova, parasites, and occult blood. Stool Cl was 120 mmol/L with zero urine Cl and K. Chest X-ray and ECG were normal. Abdominal and pelvic ultrasound did not show abnormality except for bilateral small kidneys at 8 cm with thin and echogenic cortex and few foci of papillary calcifications which were confirmed by CT scan examination (Figure 1). He was treated initially with intravenous KCl till K was >4 then with normal saline till establishing the euvolumic state. Subsequently, he required high salt and water with oral K supplementation (600 mg X2). Omeperazole 20 mg was added to protect KCl gastritis. His high uric acid was controlled with allopurinol 300 mg daily. With such measures, his electrolytes were corrected and serum creatinine decreased to 420 umol/L. On follow-up, he remained asymptomatic and his serum creatinine remained <500 umol/L for 1 year.

## 3. Discussion

Patients with CCLD have chronic watery diarrhea with high fecal Cl (>90 mmol/L), low K, and metabolic alkalosis with low urinary Cl and K [4]. Diagnosis is usually suspected with fetal abdominal distension and ultrasound features of polyhydramnios, hyperechoic bowel loops, honey comb appearance of the bowel, and dilated bowel loops with normal peristalsis [8]. Its differential diagnosis include Hirschsprung's disease, meconium ileus, anal atresia, and volvulus [9]. However, the characteristic laboratory findings assist in diagnosis and avoidance of unnecessary surgical interventions. Interestingly, it is the only diarrhea that produces metabolic alkalosis rather than metabolic acidosis. It can be differentiated from Barter's disease by having severe watery diarrhea, dehydration, failure to thrive, abdominal distention, and its electrolyte abnormalities. Contrary to Barter's, urine K is low with high Cl and its ease of correction of hypo K. Early diagnosis and treatment, with adequate salt and K, are essential for normal growth and development and prevention of other severe complications. The optimal dosage of electrolytes are Na 6 mmol/kg/day, K 6.5 mmol/kg/day, and Cl ranging from 12.5 mmol/kg/day, mmol/kg/day in infants, and less in older patients [5, 7]. The long-term disease complications have been attributed to chronic hypovolemia-induced nephrocalcinosis [10]. Virtually the majority of filtered Ca is absorbed passively in the paracellular route in the proximal convoluted tubules and ascending limb of Henle parallel to sodium. Only 10% is absorbed inversely to Na in the distal nephron [11]. Hence, with severe volume depletion, reabsorption of Ca is impaired leading to urine supersaturation and crystal precipitation with papillary calcification and nephrocalcinosis as in our patients. Moreover, maltreated gout, with NSAIDs, was a contributing factor in his papillary renal disease [12]. In conclusion; patients with CCLD should be adequately treated with salt and K supplementation and avoid NSAIDS for their associated gout.
